# Is the psychological stress related to the impact of IVF/ICSI and the INVO intravaginal device significantly different?

**DOI:** 10.5935/1518-0557.20140090

**Published:** 2014

**Authors:** Jimmy Portella, Soledad Sepulveda, Gustavo F Gonzales, Luis Guzmán

**Affiliations:** 1 PRANOR Assisted Reproduction Group, San Isidro, Lima, Peru; 2 Laboratory of Endocrinology and Reproduction, Department of Biological and Physiological Sciences, Faculty of Sciences and Philosophy, Alberto Cazorla Tálleri, Universidad Peruana Cayetano Heredia, Lima, Peru

Dear Editor-in-Chief:

Our group carefully read the article published by Vieira and Colucci (2013) in your prestigious journal. We agree that any assisted reproductive technique causes stress to the couple and that this stress is higher in women in comparison with men (El Kissi *et al*., 2013). However, in the mentioned paper, we found several remarkable mistakes, which resulted in conclusions that were not supported by the data.

First, the reported results have a serious methodological bias because the study was not randomized. Additionally, because the baseline patient characteristics were not presented, we do not know whether the groups (IVF-ICSI treatment and INVO treatment) were comparable. The psychological characteristics of the patients before ART were not shown.

Second, the study did not provide any power analysis, and more importantly, no statistical comparisons were performed. This is the main problem, as the conclusions were based on different responses to stress in patients treated using either INVO or ICSI. However, the row data included in the article support statistical analysis using Fisher’s exact test; if the authors had performed such an analysis, no differences (*P*=0.5273) (http://www.graphpad.com/quickcalcs/contingency2/) would have been observed between the studied groups. Consequently, no statement arguing for differences between the two techniques can be made.

Finally, the authors did not mention the clinical outcomes of the patients who underwent either ICSI or INVO treatment.
